# Frequency and Characteristics of Familial Melanoma in Spain: The FAM-GEM-1 Study

**DOI:** 10.1371/journal.pone.0124239

**Published:** 2015-04-13

**Authors:** Iván Márquez-Rodas, Manuel Martín González, Eduardo Nagore, Cristina Gómez-Fernández, Jose Antonio Avilés-Izquierdo, Cayetana Maldonado-Seral, Virtudes Soriano, Margarita Majem-Tarruella, Virginia Palomar, Rocio Maseda, Alfonso Martín-Carnicero, Teresa Puertolas, Elena Godoy, Pablo Cerezuela, Maria Ochoa de Olza, Begoña Campos, Elisabeth Perez-Ruiz, Ainara Soria, Irene Gil-Arnaiz, Maria Gonzalez-Cao, Elisa Galvez, Ana Arance, Joaquin Belon, Luis de la Cruz-Merino, Salvador Martín-Algarra, Spanish Multidisciplinary Group of Melanoma (GEM)

**Affiliations:** 1 Servicio de Oncología Médica, Instituto de Investigación Sanitaria Gregorio Marañón, Madrid, Spain; 2 Servicio de Dermatología, Hospital Ramón y Cajal, Madrid, Spain; 3 Servicio de Dermatología, Instituto Valenciano de Oncología, Valencia, Spain; 4 Servicio de Dermatología, Hospital La Paz, Madrid, Spain; 5 Servicio de Dermatología, Instituto de Investigación Sanitaria Gregorio Marañón, Madrid, Spain; 6 Servicio de Deramtología, Hospital Universitario Central de Asturias, Oviedo, Spain; 7 Servicio de Oncología Médica, Instituto Valenciano de Oncología, Valencia, Spain; 8 Servicio de Oncología Médcica, Hospital de Sant Pau, Barcelona, Spain; 9 Servicio de Oncología Médica, Hospital General de Valencia, Valencia, Spain; 10 Servicio de Oncología Médica, Hospital de San Pedro, Logroño, Spain; 11 Servicio de Oncología, Hospital Universitario Miguel Servet, Zaragoza, Spain; 12 Servicio de Dermatología, Hospital de Cabueñes, Gijon, Spain; 13 Servicio de Oncología Médica, Hospital General Universitario Santa Lucia, Cartagena, Spain; 14 Servicio de Oncología Médica, Instituto Catalan de Oncología, Hospitalet, Spain; 15 Servicio de Oncología Médica, Hospital Lucus Augusti, Lugo, Spain; 16 Servicio de Oncología Médica, Hospital Costa del Sol, Marbella, Spain; 17 Servicio de Oncología Médica, Hospital Ramón y Cajal, Madrid, Spain; 18 Servicio de Oncología Medica, Hospital Reina Sofía, Tudela, Spain; 19 Servico de Oncología Médica, Instituto Dexeus, Barcelona, Spain; 20 Servicio de Oncología Médica, Hospital de Elda, Alicante, Spain; 21 Servicio de Oncología Medica, Hospital Clinic, Barcelona, Spain; 22 Servicio de Oncología Médica, Clínica Oncogranada, Granada, Spain; 23 Servicio de Oncología Médica, Hospital Virgen de la Macarena, Sevilla, Spain; 24 Departamento de Oncología, Clínica Universidad de Navarra, Pamplona, Spain; University of Tennessee, UNITED STATES

## Abstract

**Introduction:**

Familial history of melanoma is a well-known risk factor for the disease, and 7% melanoma patients were reported to have a family history of melanoma. Data relating to the frequency and clinical and pathological characteristics of both familial and non-familial melanoma in Spain have been published, but these only include patients from specific areas of Spain and do not represent the data for the whole of Spain.

**Patients and methods:**

An observational study conducted by the Spanish Group of Melanoma (GEM) analyzed the family history of patients diagnosed with melanoma between 2011 and 2013 in the dermatology and oncology departments.

**Results:**

In all, 1047 patients were analyzed, and 69 (6.6%) fulfilled criteria for classical familial melanoma (two or more first-degree relatives diagnosed with melanoma). Taking into account other risk factors for familial melanoma, such as multiple melanoma, pancreatic cancer in the family or second-degree relatives with melanoma, the number of patients fulfilling the criteria increased to 165 (15.8%). Using a univariate analysis, we determined that a Breslow index of less than 1 mm, negative mitosis, multiple melanoma, and a history of sunburns in childhood were more frequent in familial melanoma patients, but a multivariate analysis revealed no differences in any pathological or clinical factor between the two groups.

**Conclusions:**

Similar to that observed in other countries, familial melanoma accounts for 6.6% of melanoma diagnoses in Spain. Although no differences in the multivariate analysis were found, some better prognosis factors, such as Breslow index, seem more frequent in familial melanoma, which reflect a better early detection marker and/or a different biological behavior.

## Introduction

Melanoma risk is determined by several factors, including sun exposure (for cutaneous melanoma), individual phenotype (phototype, presence of multiple and/or atypical nevi), and familial background. Rare genetic conditions such as xeroderma pigmentosus or hereditary retinoblastoma increase the risk for cutaneous melanoma. In a recent meta-analysis, the presence of familial aggregation of melanoma is estimated to account for 1.3–15.8% of melanoma cases, depending on the series and the countries studied [1]. Approximately 25% of these familial cases can be explained by germline mutations in cyclin-dependent kinase inhibitor 2A (*CDKN2A*), the most frequent gene mutated in familial melanoma, revealing that the genetic explanation for the majority of cases remains elusive [2,3]. Patients with *CDKN2A* mutations have a high risk of developing melanoma, as well as other tumors, the most common of which is pancreatic cancer [4]. Genetic counseling for these patients is under debate, since the real impact of prevention or early diagnosis remains unclear [5,6]

Familial melanoma is generally defined as the occurrence of melanoma in two or more first-degree relatives (in areas of heavy sun exposure, such as Australia it must be three first degree relatives) [7,8]. However, other features are considered by other researchers: both first-degree and second-degree; presence in the family of pancreatic cancer; and the presence in the same patient of multiple melanoma [2,3].

Since melanoma is a malignant tumor with one of the fastest growing incidence rates in the Western world, knowing the general epidemiological landscape, including the proportion of high-risk melanoma families, is of interest, particularly in terms of prevention, early detection, and design of public health plans.

Currently, no studies have analyzed the frequency of familial melanoma cases in Spain. Since these data are lacking, the Spanish Multidisciplinary Group of Melanoma (GEM) designed the FAM-GEM-1 study, with the aim of describing the frequency and characteristics of familial melanoma in Spain in a representative population sample.

The principal objectives of this study were to calculate the frequency of familial melanoma and to describe the clinical and pathological characteristics of familial and non-familial melanoma patients. The secondary objectives were to analyze potential differences between classic familial and non-familial melanoma patients and to calculate the frequency of patients with non-classic melanoma risk factors (multiple melanoma, melanoma and pancreatic cancer in the family, melanoma in second-degree relatives) distinct from those patients with more classic indications of familial melanoma.

## Patients and Methods

FAM-GEM-1 is an observational, national registry study. All GEM-associated investigators were invited to participate in this study. To meet the inclusion criteria patients must have been ≥18 years old, diagnosed with melanoma (both incident and prevalent cases) and have signed informed consent. The inclusion period ranged from October 2011 to October 2013. A questionnaire was completed by the patients’ attending physician and included data regarding personal, phenotypical, pathological, and familial features. A pathology report from the patient was mandatory; pathology reports from relatives were not mandatory, but recommended. Patients with familial melanoma were asked if the melanoma of their relative or relatives was diagnosed before their own diagnosis.

### Definitions

Familial melanoma was defined as the presence of two or more first-degree relatives with melanoma. Multiple melanoma was defined as the presence in the same patient of two or more invasive melanomas (independent of the histological subtype and the time of appearance) and no incidences of familial melanoma. Pancreatic and melanoma in the family was defined as the presence of pancreatic cancer and no incidences of familial melanoma. Second-degree melanoma was defined as the occurrence of melanoma in at least one second-degree relative and no incidences of familial melanoma.

### Statistical analysis

Fisher exact test or unpaired Student *t* test were performed for categorical or continuous variables comparison, respectively. A p-value <0.05 was considered statistically significant. Univariate and multivariate analyses were performed to determine if independent variables were associated with familial melanoma or non-classic familial melanoma. A p-value<0.25 in the univariate analysis for categorical variables was used to select variables of interest for the multivariate analysis. SPSS v 21 program (IBM Corporation) was used to perform the analyses.

Investigation ethical committee review board from Hospital General Universitario Gregorio Marañon (central site) approved the study. Approval from local participant institutions was obtained at each site according to Spanish regulation. This study was conducted in accordance with the ethical principles of the Declaration of Helsinki. All the participants provided their written consent to participate in this study.

## Results

### Primary objectives: frequency and characteristics of familial melanoma

From October 2011 to October 2013, 1047 consecutive patients, from 10 different Spanish regions (Andalucía, Aragon, Asturias, Cataluña, Comunidad Valenciana, Galicia, Madrid, Murcia, Navarra, and La Rioja) were recruited to participate in the study (Table 1). In all, 685 (65.4%) and 362 (34.6%) patients were recruited in the dermatology and medical oncology departments, respectively. Data were collected by 25 investigators from 21 different institutions.

**Table 1:** Distribution of familial melanoma across different regions in Spain.

SPAIN’S REGION	FAMILIAL MELANOMA RATIO (%)
**GALICIA (NORTH-WEST)**	1/19 (5.3)
**ASTURIAS (NORTH-WEST)**	2/66 (3)
**NAVARRA (NORTH)**	6/70 (8.6)
**ARAGON (NORTH)**	1/30 (3.3)
**MADRID (CENTER)**	41/480 (8.5)
**LA RIOJA (NORTH)**	1/32 (3.1)
**CATALUÑA (NORTH-EAST)**	4/64 (6.3)
**VALENCIA (EAST)**	11/232 (4.7)
**MURCIA (SOUTH-EAST)**	0/26 (0)
**ANDALUCIA (SOUTH)**	2/28 (7.1)

In all, 69 patients (6.6%) fulfilled the criteria for familial melanoma. Table 2 describes the clinical, pathological, and familial characteristics of the population, and these variables were compared using univariate analysis.

**Table 2:** Clinical and pathological characteristics of familial and non-familial melanoma patients.

CLINICAL/PATHOLOGICAL CHARACTERISTIC	FAMILIAL MELANOMA N (%) 69/1047 (6,6)	NON FAMILIAL MELANOMA N(%) 978/1047 (93,4)	*p* VALUE
AGE mean (SD)	52.5 (15.4)	55.2 (16.3)	0.1824
AGE UNDER 50	29/69 (42)	357/978 (36.5)	0.3681
SEX (MALE)	40/69 (58)	489/978 (50)	***0*.*2145***
PHOTOTYPE I-II	32/68 (47.1)	482/971 (49.6)	0.7081
RED-BLOND HAIR	17/68 (25)	191/963 (19.8)	0.3471
FAIR EYES COLOUR	24/68 (35.3)	363/968 (37.5)	0.7958
FRECKLES	17/67 (25.4)	308/960 (32.1)	0.2795
MULTYPLE NEVI	18/67 (26.9)	195/955 (20.4)	***0*.*2143***
INTERMITENT SUN EXPOSURE (SUNBED INCLUDED)	39/69 (56.5)	560/978 (57.3)	0.9006
CHRONIC SUN EXPOSURE	16/69 (23.2)	189/978 (19.3)	0.4337
SUN BURNS IN CHILDHOOD	51/66 (77.3)	580/962 (60.3)	***0*.*0059***
MELANOMA IN PREVIOUS NEVUS	22/65 (33.8)	265/883 (30)	0.5759
MULTIPLE MELANOMA	6/69 (8.7)	35/978 (3.6)	***0*.*0471***
NON-SKIN MELANOMA (INCLUDING ACRAL)	7/69 (10.1)	112/978 (11.5)	0.8466
IN SITU MELANOMA	8/53 (15.1)	95/762 (12.5)	0.5254
BRESLOW (mm), mean (SD)	1.55 (1.77)	2.27 (2.68)	0.0544
BRESLOW	>1 mm 23/53 (37.7)	>1 mm: 459/762 (61.2)	***0*.*0022***
	<1 mm:30/53 (62.3)	<1mm: 303/762 (39.8)	
ULCERATION	Positive: 11/58 (19)	Positive: 185/808 (23)	0.6259
	Negative: 47/58 (81)	Negative: 623/808 (77)	
MITOSIS	Positive: 18/50 (36)	Positive: 356/647 (65)	***0*.*0118***
	Negative: 32/50 (64)	Negative: 291/647 (45)	
NODE STATUS	Positive: 14/68 (20.6)	Positive: 278/944 (29.5)	***0*.*1291***
	Negative: 54/68 (79.4)	Negative: 666/944 (70.5)	
METASTASES	Yes: 6/69 (8.7)	Yes: 150/978 (15.3)	***0*.*1619***
	No: 63/69 (91.3)	No: 828/978 (84.7)	
BRAF MUTATED	5/7 (71.4)	59/114 (21.8)	0.4449
OTHER TUMOR (NON-SKIN)	6/69 (8.7)	87/978 (8.9)	1
PANCREATIC CANCER IN FAMILY (FIRST & SECOND DEGREE)	4/69 (5.8)	37/973 (3.8)	0.3422
OTHER TUMORS IN FAMILY (FIRST DEGREE, NON-SKIN)	30/69 (43.5)	440/978 (45)	0.9005

Bold and italic p-values indicate those variants chosen for the multivariate analysis (only categorical).

Forty-seven familial melanoma patients (68.1%) reported that their melanoma was diagnosed after the diagnoses of their relative or relatives, while 17 patients (24.6%) described their diagnosis as occurring prior to that of their relative or relatives, and 5 patients (0.5%) could not report it.

### Secondary objectives

#### Frequency of other hereditary melanoma risk factors

Taking into account other melanoma risk factors, 96 patients (9.2%) fulfilled one of the following risk criteria: 24 patients (2.3%) reported occurrence of melanoma in second-degree, but not first-degree relatives; 32 patients (3.1%) were diagnosed with multiple melanoma; 35 (3.3%) reported pancreatic cancer in the family; and 5 (0.5%) fulfilled more than one of these criteria.

### Multivariate analysis for familial melanoma

In order to investigate if there were any independent clinical or pathological characteristics described in the univariate analysis that associated with familial melanoma, a multivariate binary logistic regression analysis was conducted. Variables chosen included those whose p values in the univariate analysis were <0.25 (Table 2). Neither sex, multiple nevi, sunburn in childhood, multiple melanoma, Breslow, mitosis, node status, nor metastases were associated with familial melanoma (Table 3). A trend (p = 0.097) was detected between multiple and familial melanoma.

**Table 3:** Multivariate analysis findings for the association between familial melanoma and variables of interest.

CLINICAL/PATHOLOGICAL CHARACTERISTIC	OR for familial melanoma (95%CI)	*p* VALUE
**SEX (MALE)**	1.41 (0.7–2.87)	0.340
**MULTYPLE NEVI**	1.29 (0.53–3.14)	0.576
**SUN BURNS IN CHILDHOOD**	1.57 (0.71–3.5)	0.267
**MULTIPLE MELANOMA**	2.64 (0.84–8.33)	0.097
**BRESLOW <1 mm**	1.65 (0.7–3.84)	0.25
**NO MITOSIS**	0.78 (0.33–1.83)	0.564
**NEGATIVE NODES**	0.97 (0.4–2.32)	0.938
**NO METASTASES**	1.08 (0.31–3.81)	0.906

OR = Odds Ratio.

## Discussion

In this national registry study, with a representative sample of the Spanish population, we found a 6.6% frequency of familial melanoma, defined as the presence of two or more first-degree relatives with melanoma. After expanding the definition to less restrictive criteria, as proposed by other authors [2], the frequency of familial melanoma patients increased to 15.8%.

There are many limitations of this study that must be taken into account in order to properly interpret the results. First, the study aimed to cover all Spanish territory, but we do not have patient data from 7 of the 17 regions that constitute the Spanish territory. In addition, Madrid (Center) and Valencia (East) contribute to 712 of the 1047 patients, which is more than two thirds. Data from more areas with heavy sun exposure, such as the Canary Islands (South) and the Balearic Islands (East), would have been of interest to complete the epidemiological picture.

Second, a possible selection bias could not be excluded, even though the study included all patient diagnosed as having melanoma. Our result of 6.6% is close to the frequency described in meta-analyses [1], suggesting that the possibility of this bias is less probable.

Third, a melanoma pathology report for relatives was not mandatory, but recommended. This again could be a source of bias, especially since the presence of skin diseases can usually be confounded. For this reason, our study design limited the definition of familial melanoma only to patients having first-degree relatives diagnosed with melanoma, in order to avoid this bias. However, we believe that these results can be extrapolated to the real-world practice setting, where the pathology report of a given relative is not always available.

Notably, Madrid (Center) and Navarra (North) had almost double the frequency of familial melanoma (8.5% and 8.6%, respectively) as did Valencia (4.7%), the second region in representation. This finding can be explained plausibly using results from a previous work by Nagore et al. [8], who found a similar proportion of familial melanoma in Valencia (4.3%), in a single institution study, supporting that, at least in this region, the proportion is similar to that previously described. Some studies have appointed that there is somehow a higher proportion of inbreeding in Spain, especially in rural areas [9]. The highest ratio of familial melanoma was in Navarra (north), but also in Madrid (center), being almost the same in both cases. However, Madrid, the capital of Spain, has a high heterogeneity in its inhabitant’s origins. Thus, we believe that there is not a potential influence of inbreeding in our results.

Familial melanoma patients had no significant clinical or pathological differences in the multivariate analysis compared with that for sporadic melanoma patients. However, univariate analysis findings require further discussion.

A higher frequency of multiple melanoma was found in patients with familial melanoma. The disparity between these data and those generated by the multivariate analysis could be owing to the small number of familial melanoma cases registered, even though this represents a global sample, as indicated by the clear association between the presence of multiple melanoma and germ-line mutations in *CDKN2A*, as widely described in the literature [10,11]. Recent studies have reported that relatives of patients with multiple melanoma have a higher risk for the developing melanoma themselves [12], underlining the importance of the multiple melanoma in the familial melanoma context.

A surprising finding was that familial melanoma seemed to have better pathological prognosis factors than sporadic melanoma, including lower Breslow indexes, less positive mitoses, and a trend in less positive lymph nodes. However, it is difficult to assess if these findings reveal a different biological behavior. Moreover, in addition to the multivariate analysis, two clues described in our work may refute the idea that familial melanoma cases have better prognosis than sporadic melanoma. First, patients with familial melanoma were asked if his or her melanoma was diagnosed before or after their own relatives’ diagnoses. In all, 68.1% described their diagnosis as occurring after their relatives’, suggesting that a greater concern about the disease led to an earlier diagnosis. Second, familial melanoma patients reported more childhood sunburns than did sporadic melanoma patients (again not confirmed in the multivariate analysis). These reports could be a result of memory bias of the part of patients expressing concern for the cause of their melanoma (i.e., if a patient were to associate childhood sunburn with the cause of their melanoma and remain cognizant of this fact out of concern for other family members). In support of this, proper counseling and genetic testing among healthy relatives of melanoma patients has been reported to lead to an increase in self-skin examinations [5] and sunburn prevention [13]. This suggests that people who have experienced melanoma in their families have an increased awareness of the disease, thus explaining in part the earlier diagnoses.

One of the factors that must be taken into account when interpreting the findings of our work is that no central pathology review was carried out. This could be a source of bias, since the inter-center variability in the assessment of pathological factors cannot be excluded. It would have been interesting to analyse other pathological factors of growing interest and potential differential prognosis. For example, paucity of tumor infiltrating lymphocytes (TILs) seems to be associated with worse survival outcomes and with a higher frequency of sentinel node posititivity [14]. One factor of special interest for our research area would be melanin pigmentation. Although melanin protects against UV radiation, it is described that melanogenesis could affect the metastatic potential of melanoma, especially in advanced stages. The increase in melanin in pigmented cells lead to changes in cytoskeleton that would make melanoma cells easier to infiltrate and spread to distant organs [15]. Thus, other non-classical factors should be taken in consideration in the pathology report. However, in our present work, none of these factors were analysed. The reason was that no central pathological report was carried out, and findings from local report were used for the univariate and multivariate analysis for prognostic factors. We chose those strongly and widely accepted as prognostic (Breslow, mitotic rate, ulceration) in order to ameliorate the potential inter-center variability with other pathological factors.

Finally, carriers of a *CDKN2A* mutation have an increased risk for different tumors, with pancreatic cancer being the most frequently reported, and more recently, smoking-related tumors [16,17]. In this study, we did not detect a higher frequency of other non-skin tumors in either melanoma patients or their first-degree family members. In the case of pancreatic cancer, a trend was observed toward a higher frequency in cases of familial melanoma, but this trend was not statistically significant. It is important to note that the above-mentioned studies were focused on *CDKN2A* mutation carriers, who account for only approximately 25% of familial melanoma cases [3].

In conclusion, we can say that in Spain, according to a representative sample, 6.6% of melanoma diagnoses can be considered familial melanoma. Identification of these patients and referral of the families to cancer genetic counseling and specialized dermatologists must be included in the global management of melanoma patients. Further studies exploring molecular and genetic differences between familial and non-familial melanoma are needed for a better knowledge and management of familial melanoma.
